# P-990. Inequities in Testing for Infants Perinatally Exposed to Hepatitis C Viral Infections — Tennessee, 2018–2023

**DOI:** 10.1093/ofid/ofae631.1180

**Published:** 2025-01-29

**Authors:** Christine M Thomas, Heather Wingate, Shamia Roberts, Lindsey Sizemore, Mary-Margaret A Fill, Timothy F Jones, William Schaffner, John R Dunn

**Affiliations:** Tennessee Department of Health, Nashville, Tennessee; Tennessee Department of Health, Nashville, Tennessee; Tennessee Department of Health, Nashville, Tennessee; Tennessee Department of Health, Nashville, Tennessee; Tennessee Department of Health, Nashville, Tennessee; Tennessee Department of Health, Nashville, Tennessee; Vanderbilt University Medical Center, Nashville, Tennessee; Tennessee Department of Health, Nashville, Tennessee

## Abstract

**Background:**

National guidelines recommend testing infants perinatally exposed to hepatitis C virus (HCV). We sought to examine sociodemographic factors associated with HCV testing and infection among Tennessee infants.

Comparison of Testing Practices for Infants Perinatally Exposed to HCV by Sociodemographic Characteristics — Tennessee, January 2018–July 2020

**Figure:**
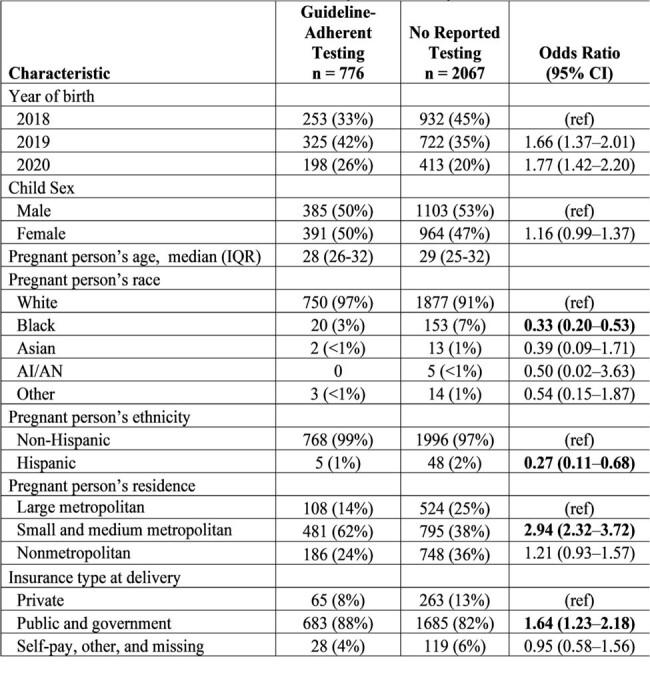

1 Percentages might not add up to 100% because of missing data.

2 This analysis excludes 177 infants with HCV testing that did not adhere with guidelines.

**Methods:**

The Tennessee Department of Health conducts active perinatal HCV surveillance by requesting that clinicians report all HCV test results for perinatally exposed infants. For infants born during January 2018–July 2020, we linked birth certificate sociodemographic information with HCV test results as of July 31, 2023. We defined HCV RNA testing at 2–36 months or antibody testing at 18–36 months of age as guideline-adherent; testing conducted outside these ages as not guideline adherent. In Tennessee, perinatal hepatitis C is considered confirmed if positive RNA testing at 2–36 months, probable if positive antibody testing only at 18–36 months, and excluded if negative RNA testing at 2–36 months or negative antibody testing at ≤ 36 months of age. We calculated odds ratios (OR) to identify sociodemographic factors associated with guideline-adherent testing.

**Results:**

Among 3,020 infants perinatally exposed to hepatitis C, 776 (26%) had guideline-adherent HCV testing, 177 (6%) had testing that was not guideline adherent, and 2,067 (68%) did not have any testing reported. Infants born to Black or Hispanic persons had lower odds of guideline-adherent testing, compared with White (OR = 0.33 [95% CI: 0.20–0.53]) or non-Hispanic (OR = 0.27 [95% CI: 0.11–0.68]) persons. Infants had higher odds of guideline-adherent testing if born to a person residing in a medium or small, compared with large metropolitan areas (OR = 2.94 [95% CI: 2.32-3.72]), or if public insurance was used at birth, compared with private insurance (OR = 1.64 [95% CI: 1.23–2.18]) (Table). Of 798 infants with hepatitis C status determined by 36 months of age, 51 (6.4%) had either confirmed (n = 45) or probable (n = 6) hepatitis C.

**Conclusion:**

Most perinatally exposed infants in Tennessee were not tested for hepatitis C. Black and Hispanic infants were less often tested and residents of medium or small areas more often tested. Equitable implementation of testing practices for perinatally exposed infants is necessary to avoid compounding disparities in hepatitis C treatment.

**Disclosures:**

**All Authors**: No reported disclosures

